# Institutional environments and breakthroughs in science. Comparison of France, Germany, the United Kingdom, and the United States

**DOI:** 10.1371/journal.pone.0239805

**Published:** 2020-09-30

**Authors:** Thomas Heinze, Marie von der Heyden, David Pithan

**Affiliations:** 1 Interdisciplinary Center of Science and Technology Studies (IZWT), University of Wuppertal, Wuppertal, Germany; 2 Institute of Sociology, University of Wuppertal, Wuppertal, Germany; Lancaster University, UNITED KINGDOM

## Abstract

Scientific and/or technical breakthroughs require the exploration of novel ideas and technologies. Yet, it has not been studied quantitatively how national institutional contexts either facilitate or stifle organizational support for exploration. Available qualitative evidence suggests that institutional contexts that exert weak control over universities and research organizations strengthen their capabilities to achieve scientific breakthroughs, while contexts with strong control constrain them. The paper is based on an analysis of the population of Nobel laureates in Physics, Chemistry and Physiology or Medicine. We examine to what extent existing qualitative findings for the biomedical sciences, which are partly based on Nobel laureates in Physiology or Medicine, can be substantiated both quantitatively and across the three Nobel Prize fields of science. We find that for most of the 20^th^ century and the early 21^st^ century, countries with weak institutional control (United Kingdom, United States) have outperformed those exerting strong control (France, Germany). These results are further corroborated when controlled by population sizes and by GDP per capita. In addition, these results hold not only for the biomedical sciences, but also for Physics and Chemistry. Furthermore, countries with weak institutional control have attracted many future Nobel laureates from countries with strong environments. In this regard, the United States appears to be a particularly attractive setting for conducting innovative research, and thus has been a magnet for young and promising scientists. However, future laureates working in institutional environments exerting weak control are not faster in accomplishing their prize-winning work compared to those laureates working in more restrictive institutional settings.

## 1. Introduction

Knowledge-based organizations face the challenge of supporting researchers in their effort to successfully address scientific and technological problems. As James March [1] has argued, the returns of experimentation with novel ideas and technologies are often uncertain, distant, and negative. In contrast, the refinement and extension of existing competences and technologies yields returns that are positive, proximate, and predictable. Hence, exploration of new paths is typically at a disadvantage compared to the exploitation of existing ones. Building on March’s classical formulation, several studies have shown that the combination of both exploratory and exploitative behavior (“ambidexterity”) enhances organizational performance [2–5]. In addition, there is growing attention to the influence of environmental conditions on organizational performance [6]. In this regard, exploration has been found particularly effective in dynamic, fast-changing environments, whereas exploitation appears to excel in stable, yet highly competitive contexts [7–9].

Most of the literature on organizational learning focuses on business firms, and much less attention has been devoted to organizations that conduct scientific research, such as universities or government-funded research centers [10–12]. Yet, these literatures have not engaged with findings from historical-comparative studies on institutional settings in national research systems and their impact on organizational performance. Both limitations are addressed by the work of Rogers Hollingsworth [13–16] who studied how national institutional contexts influenced the capabilities of research organizations to achieve scientific discoveries in the biomedical science of the 20^th^ century, using major scientific awards (including the Nobel Prize in Physiology or Medicine) as indicator for scientific performance.

Hollingsworth builds on earlier work by Ben-David [17, 18] who found that the institutional setting for conducting research has significantly differed amongst large scientific nations (France, Germany, United Kingdom, United States), and that such institutional settings played a key role in these nations’ capabilities to become global leaders in science. For example, Ben-David argued that Germany ascended to global leadership during the 19^th^ century due to its decentralized and competitive university system in which scientists migrated to those places that offered them most attractive work conditions, including state-of-the-art laboratories. Both decentralization and competition (as features of the institutional environment) emerged following the university system’s expansion in the first half of the 19^th^ century, and together they effectively remedied deficiencies in German universities’ internal structures that had limited their capability to support new research fields before.

Yet, compared to the expanding North-American university system, German universities lost their hegemonic position to the United States early in the 20^th^ century. Ben-David explains this shift, firstly, by internal organizational features in North-American universities that were more conducive to the growth of new research fields (compared to those in Germany), including effective university leadership (instead of absence of such leadership), collegiality between faculty members in academic departments (instead of hierarchy between chair holders and academic staff in institutes), research-based education in graduate schools (instead of personal dependence of individual graduate students on chair holders), and scientific careers via tenure tracks (instead of absence of any career path). Secondly, Ben-David points to the more pronounced level of decentralized competition in the North-American university system, particularly with regard to public and private universities and colleges (compared to purely state-sponsored higher education in Germany).

Hollingsworth [13–16] extended Ben-David’s findings by specifying those types of control that national institutional settings exert on research organizations, including higher education: (1) whether a particular research field, such as Molecular Biology, or a particular degree program, such as Computer Science, will be established and maintained within organizational boundaries, (2) the level of intra-organizational funding for particular research fields or degree programs, and (3) the training and appointment rules for scientific or teaching staff. As Hollingsworth points out, in countries such as France and Germany, control over universities and public research organizations is exercised to a considerable extent by state ministries. Therefore, decisions regarding (1), (2), and (3) are typically made at state level, leaving little room for universities and research organizations to maneuver in different directions. For example, French universities are allowed to establish new degree programs only with ministry approval (1), and such approval is directly connected to program funding (2). Furthermore, the number of positions and salary schemes for senior academic staff are government regulated (2), and universities must recruit professorial staff from a nation-wide list of appropriate candidates (3). In contrast, universities in the United Kingdom and the United States have more organizational and managerial autonomy, with the government playing a much less restrictive role in universities’ decision making [19–21].

Most importantly, Hollingsworth argues that these three types of external institutional control are not independent but self-reinforcing in so far that strong (or weak) control in one dimension is typically accompanied by strong (or weak) control in the other ones. Therefore, institutional contexts are configured in a way that exerts *either strong or weak control*, and thus either constrain or facilitate organizational capabilities to conduct innovative research.

A *restrictive* institutional context denotes a high degree of external control over organizational structure and behavior. In such contexts, organizations are deeply rooted in their environment and lack the autonomy to engage in self-determined strategies and objectives. Hence, research organizations in strong contexts have few degrees of freedom in their decisions regarding the funding of new research fields, the appointment of new staff, or work conditions. In contrast, *conducive* institutional contexts exercise less control over embedded organizations, they generally grant more freedom to pursue diverse behavior and offer more opportunities to respond swiftly to new circumstances. Therefore, research organizations in weakly controlled contexts can establish new and promising research fields, provide additional funding fast when scientific opportunities arise, or create supportive work conditions that help scientists to achieve breakthroughs [22, 23].

Following this line of reasoning, Hollingsworth argues that institutional contexts with strong control constrain the capabilities of research organizations to achieve scientific breakthroughs, while contexts with weak control facilitate such capabilities. Examples for strong institutional control are Germany and France, whereas the United States and the United Kingdom serve as examples for weak institutional control. The latter two, and in particular the United States, show more institutional diversity (and less isomorphism) in organizing higher education and scientific research due to their relatively large private sector that competes with public universities and research organizations. According to Hollingsworth, there should be fewer scientific breakthroughs originating from either German or French research organizations than from those located in the United States or the United Kingdom.

Although Hollingsworth presented qualitative evidence based on interviews with prestigious scientists and laureates in biomedical science and including a historical case study on the Rockefeller Institute (later: Rockefeller University), his claims have not been examined quantitatively. Furthermore, it has remained unclear if his findings apply in the case of research fields outside biomedical science. Therefore, we examine to what extent Hollingsworth’s claims can be substantiated both quantitatively and across the fields of Physics, Chemistry, and Physiology or Medicine basing our research on the population of Nobel laureates between 1901 and 2017 in the respective fields.

In section 2 of this paper, we describe our data and methodology and, in section 3, we present original findings with regard the absolute and normalized counts of Nobel laureates from France, Germany, the United Kingdom, and the United States. In addition, we examine to what extent the latter two countries (UK, US) have attracted future Nobel laureates from the former two countries (FR, DE), and thus whether migration flows occur predominantly from countries with restrictive settings to countries with conducive institutional contexts. Furthermore, we probe whether scientists make their discoveries in facilitating contexts earlier than those working in restrictive settings. In section 4, finally, we discuss our results, limitations and research desiderata.

## 2. Data & methodology

The present paper draws information from a dataset that contains all Nobel laureates in Physics, Chemistry and Physiology or Medicine from 1901 to 2017 (n = 599). This data was procured primarily from the website of the Nobel Foundation. Further sources that have contributed to the dataset are outlined in [16]. The set comprises information on: (a) the year, organization, and country of both the first and the highest academic degrees (HDs), the latter typically being either a medical doctor (M.D.) or a doctor of Philosophy (Ph.D.); (b) the year, organization, and country in which each laureate performed their prize-winning work (PWR); (c) the year, organization, and country in which the laureate worked when awarded the Nobel Prize (NP); and (d) year of birth. Regarding (b), we used publicly available data from [24], double-checked it and extended it up until 2017. From this dataset, we extracted those Nobel laureates who received their highest degrees (HD) in either of the four countries: France, Germany, United Kingdom, or United States (n = 410).

We determined the absolute and relative frequencies of Nobel laureates for the three career events (HD, PWR, NP) in these countries. The distributions in HD and PWR are mapped to the time period of the corresponding NP. The information on HD and PWR figures therefore in the period when the laureate received the NP. For instance, if the NP was received in the period from 1961 to 1970, the data on HD and PWR will also appear in the period from 1961 to 1970. This allows us to directly compare the three career events and it avoids the problem of time periods which extend back into the 19^th^ century. Hence, we provide our measures using non-overlapping ten-year periods, starting in 1901 when the Nobel Prize was awarded for the first time.

Regarding international mobility, we measure the sums of laureates’ cross-country moves from HD to PWR and/or from PWR to NP, respectively. The four countries are defined according to the university or research organization in which HD, PWR, or NP occurred. Therefore, if a future laureate received their highest degree in a German university “A” (HD), moved afterwards to another university in the United States “B” to conduct their prize-winning research (PWR), but then again moved from this institution to yet another one in the United States “C”, where they received the Nobel Prize (NP), we count one move of country from Germany to the United States (HD to PWR).

In addition to [25], we compiled historical data on the annual population by country. For the United States, we used census data, as available in [26–29]; for the United Kingdom and France, we retrieved population estimates, as available in [30] and [31], respectively. Because the national borders of Germany changed considerably after the First and Second World War, we used historically relevant territories, as available in [32–34]. For example, the universities of Strasbourg (today France) and Gdansk (today Poland) were both on the territory of Imperial Germany prior to WWI. Furthermore, we used historical data on gross domestic product (GDP) per capita, as available in [35]. Finally, we double-checked our population data [26–34] with [35] and found a very high level of consistency.

## 3. Empirical results

**Fig 1:**
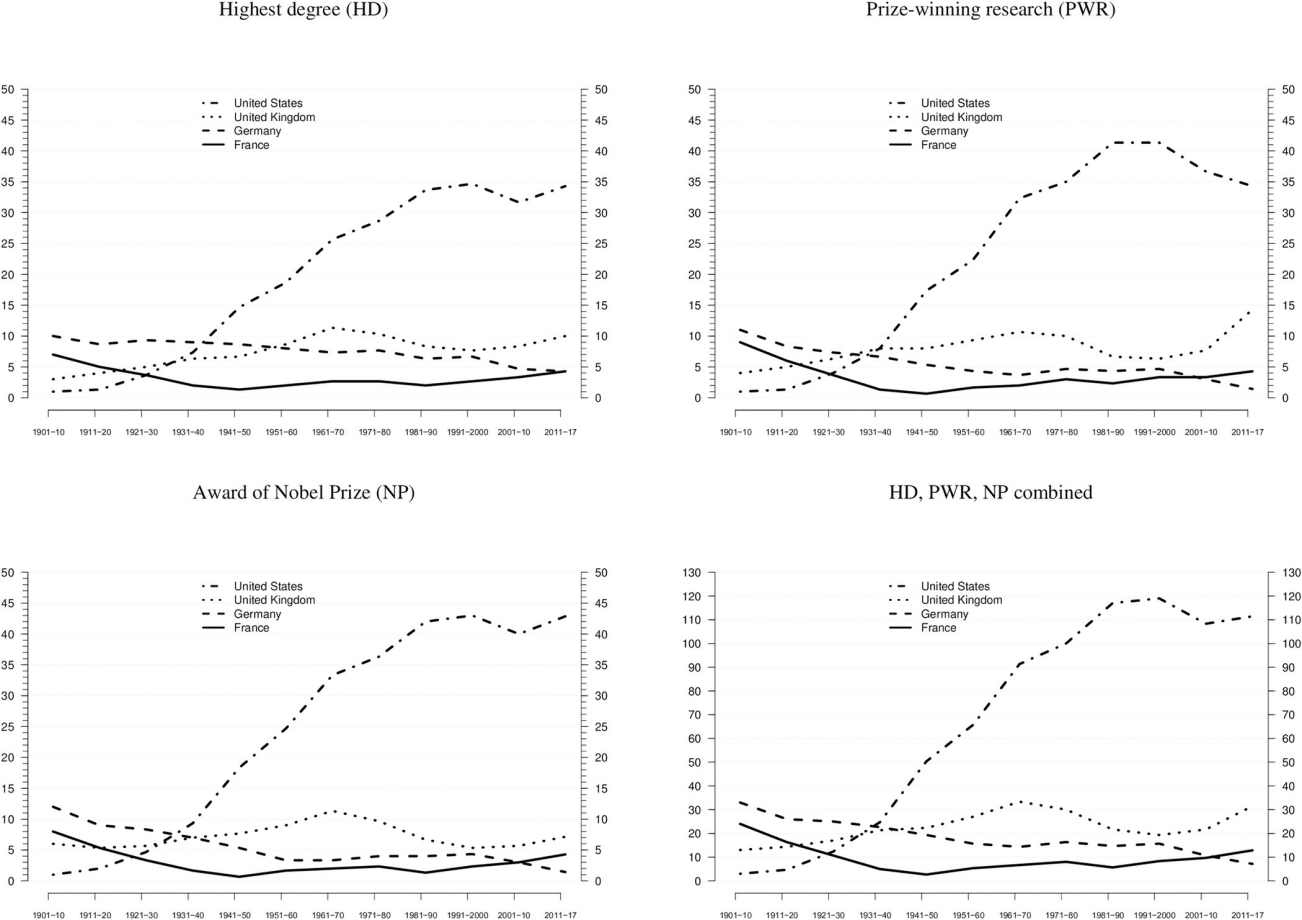
National distribution of Nobel laureates. Absolute frequencies of Nobel laureates across the four major countries, based on three career events (HD, PWR, NP). All observations refer to consecutive 10-year NP periods. Distributions in HD and PWR also are mapped onto NP periods. Lines are smoothened, using 3-period moving averages. The final period of 2011–2017 is weighted and thus comparable to earlier 10-year periods. Data from S1 Table are used.

Hollingsworth argued that national research systems with institutional environments exerting weak control generate more scientific innovations than research systems with strong control. Hence, we examined the absolute frequencies of Nobel laureates across the four major scientific nations (France, Germany, United Kingdom, United States), based on the three career events (HD, PWR, NP). As displayed in Fig 1, Germany produced most laureates in the first two decades of the 20^th^ century, whereas in the 1920, the United States started to increase its number of laureates substantially, superseding Germany in the 1930s and becoming the leading scientific nation by the 1940s. The steep slope of the United States’ curve and the long growth period (1921–2000) are remarkable. In contrast, Germany’s curve has continuously decreased over the entire observation period, while we observe a slight increase in the case of France since the 1950s. Interestingly, the United Kingdom’s curve has stayed above those of both Germany and France since the 1950s. Therefore, in absolute terms, for most of the 20^th^ century and the early 21^st^ century, countries with weak institutional environments have outperformed those with strong environments. These results provide initial quantitative support for Hollingsworth’s qualitative findings.

The strength of Hollingsworth’s argument can be further probed by two possibly competing explanatory variables: population size and GDP per capita. Taking into account the number of inhabitants rules out the possibility that institutional environments generate more innovations simply because they have larger population sizes. If a country has a large population, one could argue that its pool of scientific talent is greater, and thus can generate higher absolute quantities of innovative scientific contributions. In addition, GDP per capita is widely used in econometric analyses as an indicator of economic development, and thus for long-term national comparisons [36]. Taking into account GDP per capita rules out the possibility that institutional environments generate more scientific innovations simply because they are richer in economic resources, and thus at a more advanced level of economic development.

**Fig 2:**
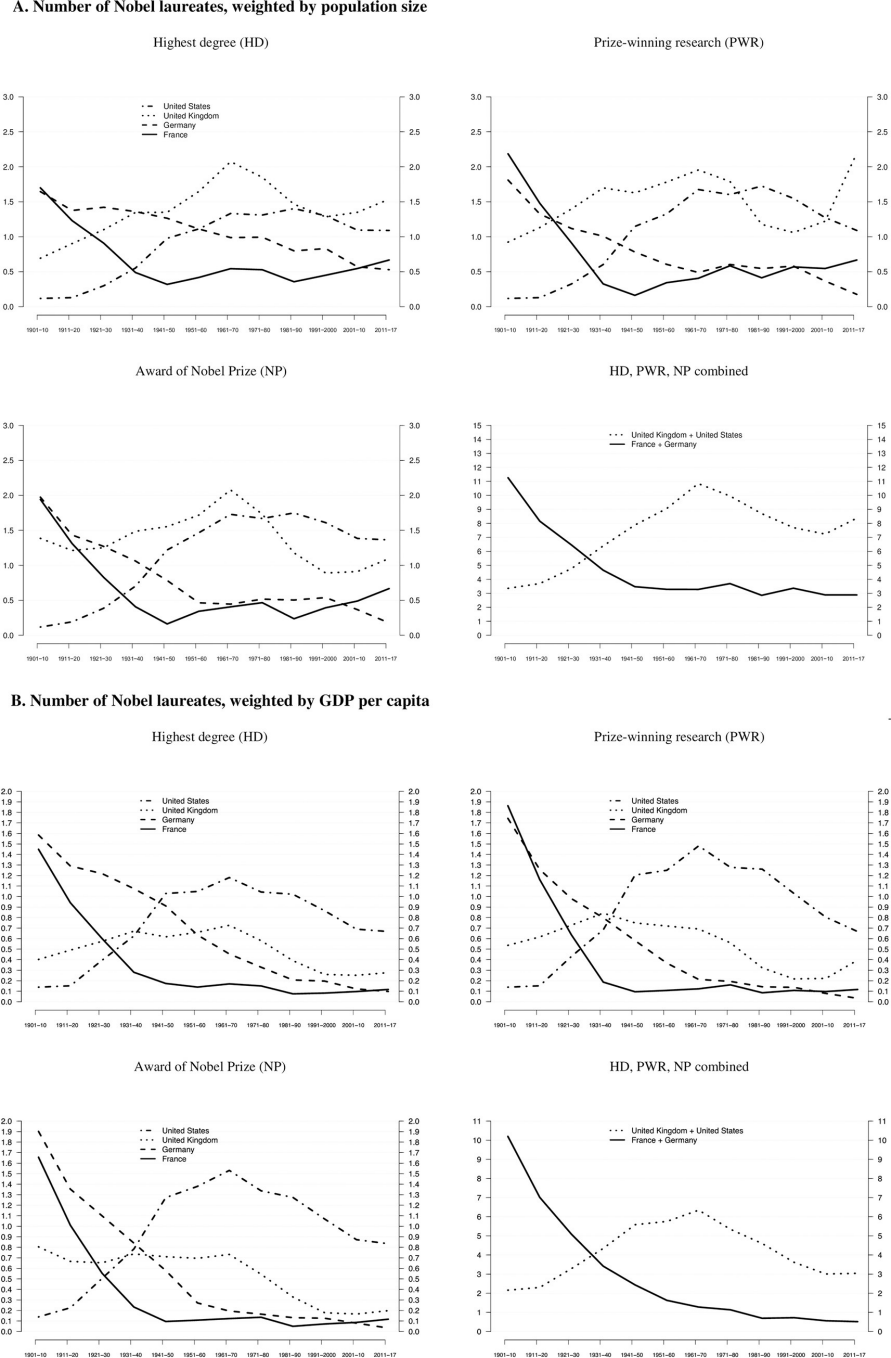
A. Number of Nobel laureates, weighted by population size. Relative frequencies of Nobel laureates, based on three career events (HD, PWR, NP), per ten million inhabitants. All observations refer to consecutive 10-year NP periods. Distributions in HD and PWR also are mapped onto NP periods. Lines are smoothened, using 3-period moving averages. The final period of 2011–2017 is weighted and thus comparable to earlier 10-year periods. Data from S3A Table are used. B. Number of Nobel laureates, weighted by GDP per capita. Relative frequencies of Nobel laureates, based on three career events (HD, PWR, NP), controlled for GDP per capita (thousand US$, in 2011 prizes). All observations refer to consecutive 10-year NP periods. Distributions in HD and PWR also are mapped onto NP periods. Lines are smoothened, using 3-period moving averages. The final period of 2011–2017 is weighted and thus comparable to earlier 10-year periods. Data from S3B Table are used.

As shown in Fig 2A, the two countries with institutional environments exerting weak control over higher education and research organizations (UK, US) yield substantially higher relative numbers of Nobel laureates than the two countries with contexts exerting strong control (FR, DE). Combining all three career events, the United Kingdom and the United States outperformed both Germany and France starting in the 1930s, reaching their peak in the 1960s. Although the relative frequency of Nobel laureates from the UK and the US combined decreased from approx. 11 laureates (1961–1970) to approx. 8 laureates (2011–2017), these two countries hosted–in relative terms–two to three times as many laureates since the mid-20^th^ century than Germany and France combined (approx. 3 laureates, 1961–2017). We conducted two sample T-Tests, both for the entire observation period 1901–2017 (UK+US = 0,719; FR+DE = 0,455; p<0,035) and the North-American hegemonic period 1961–2000 [25] (UK+US = 0,904; FR+DE = 0,354; p<0,022) that show these differences to be statistically significant at the p<0,05 level.

These results are broadly corroborated for GDP per capita, as shown in Fig 2B, including a two sample T-Tests for the North-American hegemonic period 1961–2000 (UK+US = 4,86; FR+DE = 1,01; p<0,006). However, in comparison to Fig 2A, the United States has markedly more (future) Nobel laureates, relative to GDP per capita, than the United Kingdom whose values are closer to Germany and France. Hence, controlling for economic development within the country group of weak institutional control reveals that the United States seems more effective in producing path-breaking scientific discoveries than the United Kingdom (which, in turn, outperforms Germany and France). This result is also reflected in our migration data that shows a considerable number of scientists moving from the United Kingdom to the United States during their academic career (see below). Summing up, Hollingsworth’s argument holds when population size and GDP per capita are considered, because differences between the two groups of countries persist when taking these two variables into account.

**Fig 3:**
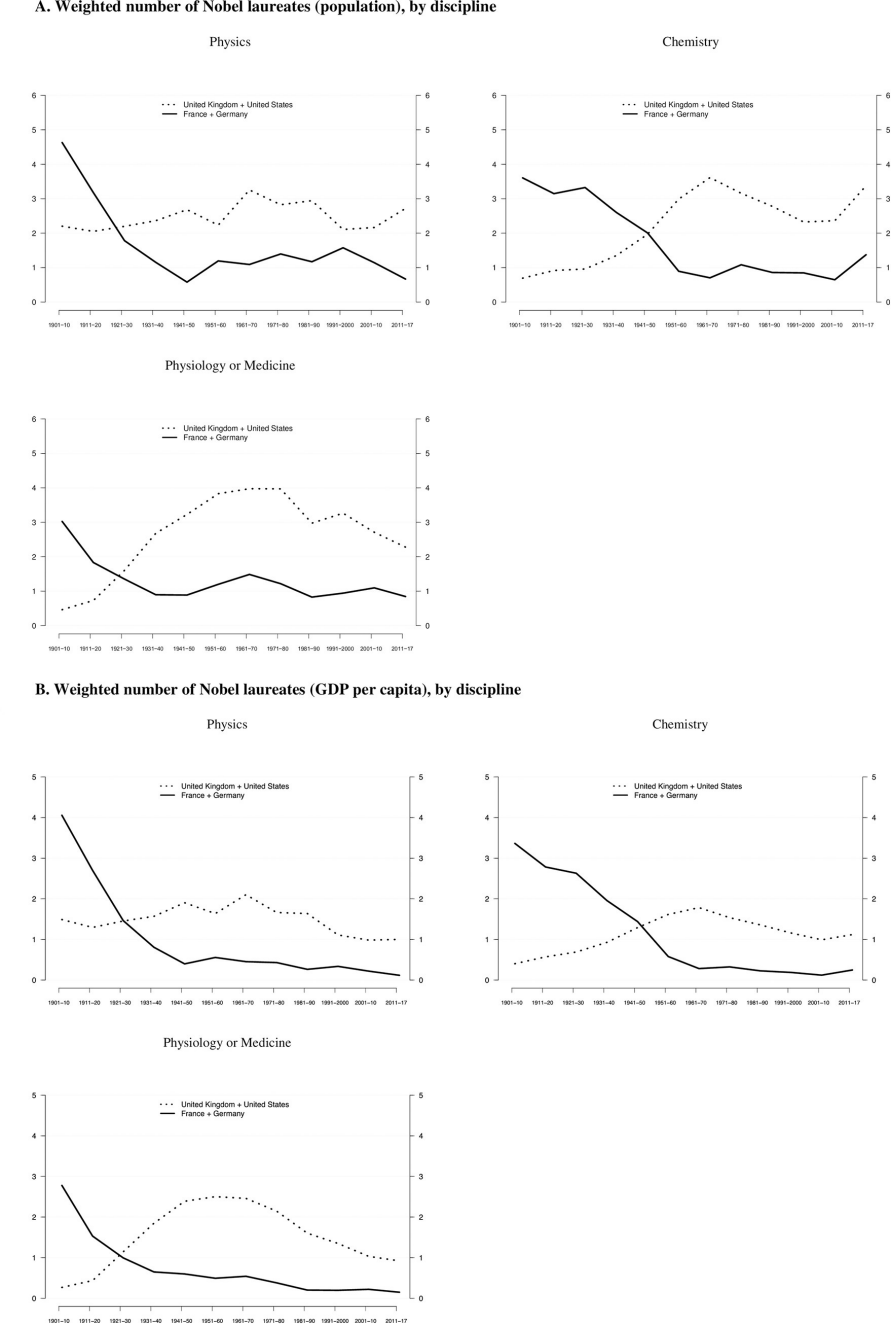
A. Weighted number of Nobel laureates (population), by discipline. Relative frequencies of Nobel laureates across countries with either weak (United Kingdom + United States) and strong (France + Germany) institutional control, based on three career events combined, per ten million inhabitants. See Fig 2A for methodical details. Data from S4A Table are used. B. Weighted number of Nobel laureates (GDP per capita), by discipline. Relative frequencies of Nobel laureates across countries with either weak (United Kingdom + United States) and strong (France + Germany) institutional control, based on three career events combined, controlled for GDP per capita (thousand US$, in 2011 prizes). See Fig 2B for methodical details. Data from S4B Table are used.

Moreover, the three disciplines are different from one another as is illustrated in Fig 3A. The gap is most pronounced in Physiology or Medicine, in which the United Kingdom and the United States have produced–controlled for population size–about three times more Nobel laureates since the 1930s than France and Germany combined. In comparison, the gap is less pronounced in Physics and Chemistry with about twice as many laureates. Furthermore, in Chemistry the United Kingdom and the United States outperformed France and Germany starting in the 1950s, and thus twenty years later than in either Physics or Medicine or Physiology (1930s). These findings are corroborated using GDP per capita as denominator, as shown in Fig 3B. Taken together, these results are noteworthy because Hollingsworth built his argument mainly on the biomedical sciences [13, 15, 16], yet they also hold–although to a somewhat lesser degree–for Physics and Chemistry. Hence, the argument regarding weak or strong institutional control can be generalized to a broader set of scientific disciplines than covered in Hollingsworth’s original formulation.

Based on the above results, we probed whether and to what extent the United Kingdom and/or the United States attracted future Nobel laureates during their career. Empirical studies have shown that the United States has been a magnet for scientists from all over the world since the mid-20^th^ century, particularly during its hegemonic period [37, 38], but it has continued to attract scientists also in the early 21^st^ century [39]. The mobility to the United States is typically motivated by better opportunities for increased productivity and/or scientific impact [40, 41] and by career advancement [42]. Therefore, it is plausible to assume that scientists educated in strongly institutionalized environments would frequently migrate to research settings in which research organizations are less determined by their institutional environment. In contrast, scientists educated in institutional environments exerting weak control over higher education and research organizations are expected to rarely move to a restrictive environment during their career.

As shown in Table 1, our data are consistent with these considerations. First, we identified 32 future Nobel laureates from France and Germany that moved to either the United Kingdom or the United States during the entire observation period, while only one Nobel laureate moved from the United States to Germany. When considering the much smaller population sizes, this migration pattern is even more pronounced than in absolute terms. Second, we find migration from the United Kingdom to the United States much more common than vice versa: three times more scientists who either received their highest academic degree in Britain or conducted their price winning work there, later moved to work in the United States. The United States appears to be a comparatively more attractive setting for conducting innovative research than the United Kingdom, although the latter is still attractive for scientists educated either in France or Germany.

**Table 1:** Cross-country mobility of Nobel laureates.

Observed Frequencies		Receiving Country			
Sending Country		France	Germany	United Kingdom	United States
	France	-	2	0	3
	Germany	2	-	10	19
	United Kingdom	0	0	-	21
	United States	0	1	7	-

Observed frequencies of (future) laureate mobility for the entire observation period 1901–2017. Mobility includes moves from HD to PWR and/or from PWR to NP.

Based on this migration pattern, we probed if institutional environments exerting weak control provided future Nobel laureates with an individual time advantage compared to scientists conducting their research in more restrictive environments. Because scientists in environments with weak institutional control have more freedom to pursue their own research goals, they may have more opportunities to conduct high-risk projects, and, thus, conduct their prize-winning research earlier in their career than laureates located in Germany or France. We measured the distance in years between laureates’ HD and their PWR. Yet, as shown in Table 2, this time lag is very similar in both institutional contexts: 13.6 years in weak ones (UK, US) and 13.7 years in strong ones (FR, DE). Consequently, there is no evidence for scientists in contexts exerting weak control to achieve innovative research more swiftly than their peers in environments with strong control. Most interesting is the fact that scientists moving from “strong” to “weak” environments do not take significantly longer to accomplish their prize-winning work (14.0 years), indicating that there is no penalty when moving to the United Kingdom or the United States (Table 2).

**Table 2:** Time lag between highest degree and prize-winning research.

		PWR			
HD		France	Germany	United Kingdom	United States
	France	13,7 (3,04)		14,0 (1,51)	
	Germany				
	United Kingdom	—		13,6 (0,78)	
	United States				

Average number of years (mean, standard deviation in parentheses) between highest degree (HD) and prize-winning research (PWR). Data covers the entire observation period. Mobility includes moves from HD to PWR and/or from PWR to NP. The bottom left cell is empty, because only one scientist moved from the United States to Germany (see Table 1). Hence, no mean value could be computed.

Together with the results presented above, we conclude that future Nobel laureates are a highly homogeneous “ultra-elite” [43] in terms of average duration of years needed to achieve scientific breakthroughs. Hence, although restrictive institutional environments lower the overall probability to accomplish scientific breakthroughs, especially because of substantial migration from countries with strong institutional environments to countries with weak institutional control, individual achievements do not take significantly longer in more restrictive settings.

## 4. Discussion

Although it is widely acknowledged that scientific and/or technical breakthroughs require organizations to support the exploration of novel ideas and technologies, it has not been studied quantitatively how national institutional contexts have shaped organizational capabilities in this regard. Available qualitative evidence, mostly based on case-studies and cross-national comparisons in the biomedical sciences, suggests that institutional contexts exerting strong control over universities and research organizations constrain their capabilities to achieve scientific breakthroughs, while contexts with weak control facilitate them. Our contribution is based on an analysis of the population of Nobel laureates in Physics, Chemistry, and Physiology or Medicine, focusing on those four countries with most contributions to scientific breakthroughs awarded with Nobel prizes between 1901 and 2017: United States, United Kingdom, Germany, and France. We examine to what extent Hollingsworth’s qualitative findings for the biomedical sciences, which build on earlier work by Ben-David, can be substantiated both quantitatively and across the three fields of science.

In absolute terms, for most of the 20^th^ century and particularly since the 1930s, but also for the early 21^st^ century, countries with weak institutional control have outperformed those with institutional contexts exerting strong control. These results are further corroborated when controlled both by population size and by GDP per capita respectively. Countries with weak institutional control (UK, US) yield substantially higher numbers of Nobel laureates than countries with strong institutional control (FR, DE). Although their share of Nobel laureates has decreased to a certain degree, both the United States and the United Kingdom hosted between twice (population size) and three times (GDP per capita) as many laureates than Germany and France. In addition, these results hold not only for the biomedical sciences, but also for Physics and Chemistry. Therefore, the argument regarding weak or strong control can be applied to a broader set of scientific disciplines than in Hollingsworth’s original formulation.

We also found that the United Kingdom and the United States attracted many future Nobel laureates from either Germany or France. In contrast, we identified only one future Nobel laureate who moved from the United States to Germany. In addition, we found that migration from the United Kingdom to the United States is more common than vice versa. Hence, the United States appears to be a particularly attractive setting for conducting innovative research, and thus is a magnet for young and promising scientists. Our findings corroborate earlier findings on the issue of academic migration to the United States [37, 39–42].

This paper provides descriptive evidence for how national research systems differ in their capability to produce scientific breakthroughs, including new theories and methods, discoveries of new materials, or new research instrumentation [44]. However, a more complete explanation would require a statistical model that includes not only the weighted number of Nobel laureates as explanatory variable, but suitable measures for the institutional environment’s influence on research organizations in different national contexts, as highlighted by Hollingsworth: (1) whether a particular research field will be established and maintained within the boundaries of a research organization, (2) the level of funding for particular research fields, and (3) the training and appointment rules for scientific staff. The paper uses Hollingsworth’s characterization [13–16], but does not measure these explanatory variables (1)–(3). Such measurements would require comprehensive collection of historical data over both the 20^th^ and 21^st^ centuries, including national and regional administrative regulation of universities and research organizations, their funding and staff structure, and also national labor law.

Also, such a statistical model would have to incorporate institutional change. First, recent bibliometric comparisons show a declining citation impact gap between the United States and other world regions since the 2000s [45–47]. This hints at a decreasing competitive advantage of the United States, and thus decreasing organizational capabilities to conduct prize-winning research. Second, despite considerable attempts at reforming European university systems, such efforts have apparently not led to deep-level changes [48–50]. Although there has been both legal and administrative change in German science policy, particularly since the 1990s, the traditionally strong role of state ministries and intermediary agencies on decisions regarding (1)–(3) has remained largely intact [48, 49, 51–53]. Similarly, French universities are regulated at the national level, most scientists are civil servants, and universities cannot negotiate employees’ salaries. Despite several legal and administrative changes in recent years, the overall state-governed architecture of the French system has not changed [54–56]. Therefore, it seems fair to say that institutional environments both in Germany and France still exercise “strong control” over research organizations. Furthermore, key features in the institutional environment of British universities have been stable, including limited state control of the academic labor market and a high level of state delegation over managerial coordination to university leadership. Yet the accountability pressure via the Research Excellence Framework (started in the mid 1980s as Research Assessment Exercise) appears to have “strengthened” the institutional environment’s influence on universities’ decisions regarding (1)–(3) [57–59]. These exemplary, mostly qualitative findings need further validation by systematic collection of historical data over both the 20^th^ and 21^st^ centuries, as mentioned above. To our knowledge, such comprehensive data are currently not available.

Further research should also focus on research systems like Japan that are outside Hollingsworth’s theoretical perspective, but which have received high-ranking scientific prizes in recent years and thrive with a rising number of Nobel laureates [25]. Another possible avenue for further research could be a broader disciplinary spectrum, including more high-ranking scientific awards in other disciplines, such as the Fields Medal in Mathematics. Also, more efforts are necessary for comparative data below the national level, particularly for countries, such as Germany, in which regions play a key regulatory and financial role in higher education and public research. One such attempt is the *autonomy scorecard* (measuring organizational, financial, staffing, and academic autonomy) of the European University Association that provides not only comparative data for France and the United Kingdom at the national level, but also data for three German Länder (North Rhine-Westfalia, Hesse, and Brandenburg) in the 2000s and 2010s [19, 60]. Although the overall scores for both financial and staffing autonomy are consistent with Hollingsworth (United Kingdom: high, France: medium-low, German Länder: medium-low), they are neither available for all German Länder nor for the United States. Finally, we believe that this paper adds a useful historical-sociological perspective to those studies that compare Europe’s (typically EU 28 plus Norway, Switzerland) research performance with that of the United States. These studies consistently find that Europe lags behind the United States in the production of high impact research [46, 47, 61–63]. Their results are broadly consistent with our findings, yet the differences between the United Kingdom on the one hand, and France and Germany on the other hand, suggest that future performance comparisons should examine the United Kingdom separately from Europe.

## Supporting information

S1 Table Number of Nobel laureates. (DOCX)

S2 Table A. Average number of inhabitants in million. S2B Table. Amount of US$ per capita, in 2011 prizes.
(DOCX)

S3 Table A. Number of Nobel laureates per ten million inhabitants. S3B Table. Number of Nobel laureates, weighted by GDP per capita.
(DOCX)

S4 Table A. Weighted number of Nobel laureates (population), by scientific discipline. S4B Table. Weighted number of Nobel laureates (GDP per capita), by scientific discipline.
(DOCX)
