# Supplementary material for: Institutional environments and breakthroughs in science. Comparison of France, Germany, the United Kingdom, and the United States
Source: PLoS One. 2020 Sep 30;15(9):e0239805. doi: 10.1371/journal.pone.0239805 (PMC7526927; doi:10.1371/journal.pone.0239805)
Supplement: S1 Table — (DOCX) [file pone.0239805.s001.docx]

S1 Table. Number of Nobel laureates

| Award Period | France | Germany | United Kingdom | United States |
| --- | --- | --- | --- | --- |
|  | Highest Degree (HD) | | | |
| 1901-1910 | 7 | 10 | 3 | 1 |
| 1911-1920 | 5 | 7 | 2 | 1 |
| 1921-1930 | 3 | 9 | 7 | 2 |
| 1931-1940 | 3 | 12 | 6 | 8 |
| 1941-1950 | 0 | 6 | 6 | 12 |
| 1951-1960 | 1 | 8 | 8 | 24 |
| 1961-1970 | 5 | 10 | 12 | 20 |
| 1971-1980 | 2 | 4 | 14 | 33 |
| 1981-1990 | 1 | 9 | 5 | 33 |
| 1991-2000 | 3 | 6 | 6 | 35 |
| 2001-2010 | 4 | 5 | 12 | 36 |
| 2011-2017 | 4 | 4 | 10 | 34 |
|  | Prize-winning research (PWR) | | | |
| 1901-1910 | 9 | 11 | 4 | 1 |
| 1911-1920 | 7 | 7 | 3 | 1 |
| 1921-1930 | 2 | 7 | 8 | 2 |
| 1931-1940 | 2 | 8 | 8 | 9 |
| 1941-1950 | 0 | 5 | 8 | 13 |
| 1951-1960 | 0 | 3 | 8 | 30 |
| 1961-1970 | 5 | 5 | 12 | 24 |
| 1971-1980 | 1 | 3 | 12 | 43 |
| 1981-1990 | 3 | 6 | 6 | 38 |
| 1991-2000 | 3 | 4 | 2 | 43 |
| 2001-2010 | 4 | 4 | 11 | 43 |
| 2011-2017 | 4 | 1 | 14 | 34 |
|  | Nobel Prize (NP) | | | |
| 1901-1910 | 8 | 12 | 6 | 1 |
| 1911-1920 | 5 | 7 | 3 | 2 |
| 1921-1930 | 3 | 8 | 7 | 3 |
| 1931-1940 | 2 | 10 | 7 | 9 |
| 1941-1950 | 0 | 3 | 7 | 16 |
| 1951-1960 | 0 | 3 | 9 | 30 |
| 1961-1970 | 5 | 4 | 11 | 28 |
| 1971-1980 | 1 | 3 | 14 | 42 |
| 1981-1990 | 1 | 5 | 4 | 39 |
| 1991-2000 | 2 | 4 | 2 | 45 |
| 2001-2010 | 4 | 4 | 10 | 45 |
| 2011-2017 | 4 | 1 | 7 | 43 |

Absolute frequencies of Nobel laureates across the three career events (HD, PWR, NP). The final period of 2011–2017 (NP) is weighted and thus comparable to earlier 10-year periods.
