# Supplementary material for: Institutional environments and breakthroughs in science. Comparison of France, Germany, the United Kingdom, and the United States
Source: PLoS One. 2020 Sep 30;15(9):e0239805. doi: 10.1371/journal.pone.0239805 (PMC7526927; doi:10.1371/journal.pone.0239805)
Supplement: S2 Table — A. Average number of inhabitants in million. S2B Table. Amount of US$ per capita, in 2011 prizes. (DOCX) [file pone.0239805.s002.docx]

S2A Table. Average number of inhabitants in million

|  | France | Germany | United Kingdom | United States |
| --- | --- | --- | --- | --- |
| 1901-1910 | 41,19 | 60,78 | 43,31 | 84,73 |
| 1911-1920 | 39,78 | 65,87 | 42,16 | 100,51 |
| 1921-1930 | 40,82 | 63,43 | 45,48 | 116,24 |
| 1931-1940 | 41,01 | 67,34 | 46,47 | 127,78 |
| 1941-1950 | 40,71 | 68,34 | 49,54 | 139,63 |
| 1951-1960 | 44,38 | 70,63 | 51,70 | 165,93 |
| 1961-1970 | 48,54 | 75,65 | 54,99 | 193,89 |
| 1971-1980 | 53,15 | 78,47 | 56,25 | 217,23 |
| 1981-1990 | 55,86 | 78,23 | 56,90 | 239,11 |
| 1991-2000 | 58,34 | 81,65 | 58,46 | 267,72 |
| 2001-2010 | 60,86 | 82,25 | 61,60 | 296,48 |
| 2011-2017* | 64,22 | 81,00 | 65,10 | 314,50 |

Data sources: [20-28]. *The final period of 2011–2017 contains values for inhabitants up until 2015.

S2B Table. Amount of US$ per capita, in 2011 prizes

|  | France | Germany | United Kingdom | United States |
| --- | --- | --- | --- | --- |
| 1901-1910 | 4831 | 6310 | 7459 | 7209 |
| 1911-1920 | 5200 | 6220 | 7984 | 8070 |
| 1921-1930 | 7253 | 7746 | 8546 | 10384 |
| 1931-1940 | 7032 | 8784 | 9160 | 9212 |
| 1941-1950 | 6032 | 8603 | 11146 | 14724 |
| 1951-1960 | 10408 | 11918 | 12167 | 17108 |
| 1961-1970 | 15488 | 18685 | 15294 | 21563 |
| 1971-1980 | 22048 | 25442 | 18973 | 27257 |
| 1981-1990 | 26298 | 30757 | 22832 | 33271 |
| 1991-2000 | 30769 | 35021 | 27973 | 40534 |
| 2001-2010 | 35726 | 39718 | 34733 | 48684 |
| 2011-2016* | 36729 | 43804 | 36081 | 51367 |

Data source: [29]. *The final period of 2011–2016 contains values for GDP per capita up until 2016.
