# Supplementary material for: Institutional environments and breakthroughs in science. Comparison of France, Germany, the United Kingdom, and the United States
Source: PLoS One. 2020 Sep 30;15(9):e0239805. doi: 10.1371/journal.pone.0239805 (PMC7526927; doi:10.1371/journal.pone.0239805)
Supplement: S3 Table — A. Number of Nobel laureates per ten million inhabitants. S3B Table. Number of Nobel laureates, weighted by GDP per capita. (DOCX) [file pone.0239805.s003.docx]

S3A Table. Number of Nobel laureates per ten million inhabitants

| Award Period | France | Germany | United Kingdom | United States |
| --- | --- | --- | --- | --- |
|  | Highest Degree (HD) | | | |
| 1901-1910 | 1,699 | 1,645 | 0,693 | 0,118 |
| 1911-1920 | 1,257 | 1,063 | 0,474 | 0,099 |
| 1921-1930 | 0,735 | 1,419 | 1,539 | 0,172 |
| 1931-1940 | 0,732 | 1,782 | 1,291 | 0,626 |
| 1941-1950 | 0,000 | 0,878 | 1,211 | 0,859 |
| 1951-1960 | 0,225 | 1,133 | 1,547 | 1,446 |
| 1961-1970 | 1,030 | 1,322 | 2,182 | 1,032 |
| 1971-1980 | 0,376 | 0,510 | 2,489 | 1,519 |
| 1981-1990 | 0,179 | 1,150 | 0,879 | 1,380 |
| 1991-2000 | 0,514 | 0,735 | 1,026 | 1,307 |
| 2001-2010 | 0,657 | 0,608 | 1,948 | 1,214 |
| 2011-2017 | 0,667 | 0,529 | 1,536 | 1,090 |
|  | Prize-winning research (PWR) | | | |
| 1901-1910 | 2,185 | 1,810 | 0,924 | 0,118 |
| 1911-1920 | 1,760 | 1,063 | 0,712 | 0,099 |
| 1921-1930 | 0,490 | 1,104 | 1,759 | 0,172 |
| 1931-1940 | 0,488 | 1,188 | 1,722 | 0,704 |
| 1941-1950 | 0,000 | 0,732 | 1,615 | 0,931 |
| 1951-1960 | 0,000 | 0,425 | 1,547 | 1,808 |
| 1961-1970 | 1,030 | 0,661 | 2,182 | 1,238 |
| 1971-1980 | 0,188 | 0,382 | 2,133 | 1,979 |
| 1981-1990 | 0,537 | 0,767 | 1,054 | 1,589 |
| 1991-2000 | 0,514 | 0,490 | 0,342 | 1,606 |
| 2001-2010 | 0,657 | 0,486 | 1,786 | 1,450 |
| 2011-2017 | 0,667 | 0,176 | 2,194 | 1,090 |
|  | Nobel Prize (NP) | | | |
| 1901-1910 | 1,942 | 1,974 | 1,385 | 0,118 |
| 1911-1920 | 1,257 | 1,063 | 0,712 | 0,199 |
| 1921-1930 | 0,735 | 1,261 | 1,539 | 0,258 |
| 1931-1940 | 0,488 | 1,485 | 1,506 | 0,704 |
| 1941-1950 | 0,000 | 0,439 | 1,413 | 1,146 |
| 1951-1960 | 0,000 | 0,425 | 1,741 | 1,808 |
| 1961-1970 | 1,030 | 0,529 | 2,000 | 1,444 |
| 1971-1980 | 0,188 | 0,382 | 2,489 | 1,933 |
| 1981-1990 | 0,179 | 0,639 | 0,703 | 1,631 |
| 1991-2000 | 0,343 | 0,490 | 0,342 | 1,681 |
| 2001-2010 | 0,657 | 0,486 | 1,623 | 1,518 |
| 2011-2017 | 0,667 | 0,176 | 1,097 | 1,363 |

Relative frequencies of Nobel laureates across the three career events (HD, PWR, NP). The final period of 2011–2017 (NP) is weighted and thus comparable to earlier 10-year periods.

S3B Table. Number of Nobel laureates, weighted by GDP per capita

| Award Period | France | Germany | United Kingdom | United States |
| --- | --- | --- | --- | --- |
|  | Highest Degree (HD) | | | |
| 1901-1910 | 1,449 | 1,585 | 0,402 | 0,139 |
| 1911-1920 | 0,962 | 1,125 | 0,250 | 0,124 |
| 1921-1930 | 0,414 | 1,162 | 0,819 | 0,193 |
| 1931-1940 | 0,427 | 1,366 | 0,655 | 0,868 |
| 1941-1950 | 0,000 | 0,697 | 0,538 | 0,815 |
| 1951-1960 | 0,096 | 0,671 | 0,658 | 1,403 |
| 1961-1970 | 0,323 | 0,535 | 0,785 | 0,927 |
| 1971-1980 | 0,091 | 0,157 | 0,738 | 1,211 |
| 1981-1990 | 0,038 | 0,293 | 0,219 | 0,992 |
| 1991-2000 | 0,097 | 0,171 | 0,214 | 0,863 |
| 2001-2010 | 0,112 | 0,126 | 0,345 | 0,739 |
| 2011-2017 | 0,117 | 0,098 | 0,277 | 0,667 |
|  | Prize-winning research (PWR) | | | |
| 1901-1910 | 1,863 | 1,743 | 0,536 | 0,139 |
| 1911-1920 | 1,346 | 1,125 | 0,376 | 0,124 |
| 1921-1930 | 0,276 | 0,904 | 0,936 | 0,193 |
| 1931-1940 | 0,284 | 0,911 | 0,873 | 0,977 |
| 1941-1950 | 0,000 | 0,581 | 0,718 | 0,883 |
| 1951-1960 | 0,000 | 0,252 | 0,658 | 1,754 |
| 1961-1970 | 0,323 | 0,268 | 0,785 | 1,113 |
| 1971-1980 | 0,045 | 0,118 | 0,632 | 1,578 |
| 1981-1990 | 0,114 | 0,195 | 0,263 | 1,142 |
| 1991-2000 | 0,097 | 0,114 | 0,071 | 1,061 |
| 2001-2010 | 0,112 | 0,101 | 0,317 | 0,883 |
| 2011-2017 | 0,117 | 0,033 | 0,396 | 0,667 |
|  | Nobel Prize (NP) | | | |
| 1901-1910 | 1,656 | 1,902 | 0,804 | 0,139 |
| 1911-1920 | 0,962 | 1,125 | 0,376 | 0,248 |
| 1921-1930 | 0,414 | 1,033 | 0,819 | 0,289 |
| 1931-1940 | 0,284 | 1,138 | 0,764 | 0,977 |
| 1941-1950 | 0,000 | 0,349 | 0,628 | 1,087 |
| 1951-1960 | 0,000 | 0,252 | 0,740 | 1,754 |
| 1961-1970 | 0,323 | 0,214 | 0,719 | 1,298 |
| 1971-1980 | 0,045 | 0,118 | 0,738 | 1,541 |
| 1981-1990 | 0,038 | 0,163 | 0,175 | 1,172 |
| 1991-2000 | 0,065 | 0,114 | 0,071 | 1,110 |
| 2001-2010 | 0,112 | 0,101 | 0,288 | 0,924 |
| 2011-2017 | 0,117 | 0,033 | 0,198 | 0,834 |

Relative frequencies of Nobel laureates across the three career events (HD, PWR, NP), controlled for GDP per capita (thousand US$, in 2011 prizes). The final period of 2011–2017 (NP) is weighted and thus comparable to earlier 10-year periods.
