# Supplementary material for: Institutional environments and breakthroughs in science. Comparison of France, Germany, the United Kingdom, and the United States
Source: PLoS One. 2020 Sep 30;15(9):e0239805. doi: 10.1371/journal.pone.0239805 (PMC7526927; doi:10.1371/journal.pone.0239805)
Supplement: S4 Table — A. Weighted number of Nobel laureates (population), by scientific discipline. S4B Table. Weighted number of Nobel laureates (GDP per capita), by scientific discipline. (DOCX) [file pone.0239805.s004.docx]

S4A Table. Weighted number of Nobel laureates (population), by scientific discipline

| Award Period | France | Germany | United Kingdom | United States |
| --- | --- | --- | --- | --- |
|  | Physics | | | |
| 1901-1910 | 3,642 | 0,987 | 1,847 | 0,354 |
| 1911-1920 | 0,503 | 1,822 | 1,898 | 0,000 |
| 1921-1930 | 1,470 | 1,104 | 1,539 | 0,516 |
| 1931-1940 | 0,000 | 0,446 | 1,937 | 0,704 |
| 1941-1950 | 0,000 | 0,439 | 1,817 | 0,573 |
| 1951-1960 | 0,000 | 0,849 | 0,967 | 2,049 |
| 1961-1970 | 1,236 | 1,058 | 0,000 | 1,289 |
| 1971-1980 | 0,000 | 0,127 | 3,200 | 2,256 |
| 1981-1990 | 0,358 | 1,406 | 0,351 | 1,380 |
| 1991-2000 | 1,371 | 0,245 | 0,000 | 1,644 |
| 2001-2010 | 0,493 | 0,851 | 1,461 | 1,484 |
| 2011-2017 | 0,667 | 0,000 | 1,536 | 1,181 |
|  | Chemistry | | | |
| 1901-1910 | 0,971 | 2,632 | 0,693 | 0,000 |
| 1911-1920 | 2,262 | 1,366 | 0,000 | 0,298 |
| 1921-1930 | 0,000 | 2,207 | 1,759 | 0,000 |
| 1931-1940 | 1,463 | 2,673 | 0,430 | 0,391 |
| 1941-1950 | 0,000 | 1,463 | 0,606 | 0,859 |
| 1951-1960 | 0,000 | 0,425 | 2,708 | 0,904 |
| 1961-1970 | 0,000 | 0,793 | 3,273 | 0,619 |
| 1971-1980 | 0,000 | 0,892 | 2,133 | 1,197 |
| 1981-1990 | 0,537 | 1,023 | 0,703 | 1,547 |
| 1991-2000 | 0,000 | 0,122 | 1,368 | 1,382 |
| 2001-2010 | 0,493 | 0,365 | 0,487 | 1,484 |
| 2011-2017 | 0,667 | 0,705 | 2,194 | 1,181 |
|  | Physiology or Medicine | | | |
| 1901-1910 | 1,214 | 1,810 | 0,462 | 0,000 |
| 1911-1920 | 1,508 | 0,000 | 0,000 | 0,099 |
| 1921-1930 | 0,490 | 0,473 | 1,539 | 0,086 |
| 1931-1940 | 0,244 | 1,337 | 2,152 | 0,939 |
| 1941-1950 | 0,000 | 0,146 | 1,817 | 1,504 |
| 1951-1960 | 0,225 | 0,708 | 1,161 | 2,109 |
| 1961-1970 | 1,854 | 0,661 | 3,091 | 1,805 |
| 1971-1980 | 0,753 | 0,255 | 1,778 | 1,979 |
| 1981-1990 | 0,000 | 0,128 | 1,582 | 1,673 |
| 1991-2000 | 0,000 | 1,347 | 0,342 | 1,569 |
| 2001-2010 | 0,986 | 0,365 | 3,409 | 1,214 |
| 2011-2017 | 0,667 | 0,176 | 1,097 | 1,181 |

Frequencies of Nobel laureates across the three career events (HD, PWR, NP) per ten million inhabitants. The final period of 2011–2017 (NP) is weighted and thus comparable to earlier 10-year periods.

S4B Table. Weighted number of Nobel laureates (GDP per capita), by scientific discipline

| Award Period | France | Germany | United Kingdom | United States |
| --- | --- | --- | --- | --- |
|  | Physics | | | |
| 1901-1910 | 3,105 | 0,951 | 1,073 | 0,416 |
| 1911-1920 | 0,385 | 1,929 | 1,002 | 0,000 |
| 1921-1930 | 0,827 | 0,904 | 0,819 | 0,578 |
| 1931-1940 | 0,000 | 0,342 | 0,983 | 0,977 |
| 1941-1950 | 0,000 | 0,349 | 0,807 | 0,543 |
| 1951-1960 | 0,000 | 0,503 | 0,411 | 1,987 |
| 1961-1970 | 0,387 | 0,428 | 0,000 | 1,159 |
| 1971-1980 | 0,000 | 0,039 | 0,949 | 1,798 |
| 1981-1990 | 0,076 | 0,358 | 0,088 | 0,992 |
| 1991-2000 | 0,260 | 0,057 | 0,000 | 1,086 |
| 2001-2010 | 0,084 | 0,176 | 0,259 | 0,904 |
| 2011-2017 | 0,117 | 0,000 | 0,277 | 0,723 |
|  | Chemistry | | | |
| 1901-1910 | 0,828 | 2,536 | 0,402 | 0,000 |
| 1911-1920 | 1,731 | 1,447 | 0,000 | 0,372 |
| 1921-1930 | 0,000 | 1,807 | 0,936 | 0,000 |
| 1931-1940 | 0,853 | 2,049 | 0,218 | 0,543 |
| 1941-1950 | 0,000 | 1,162 | 0,269 | 0,815 |
| 1951-1960 | 0,000 | 0,252 | 1,151 | 0,877 |
| 1961-1970 | 0,000 | 0,321 | 1,177 | 0,556 |
| 1971-1980 | 0,000 | 0,275 | 0,632 | 0,954 |
| 1981-1990 | 0,114 | 0,260 | 0,175 | 1,112 |
| 1991-2000 | 0,000 | 0,029 | 0,286 | 0,913 |
| 2001-2010 | 0,084 | 0,076 | 0,086 | 0,904 |
| 2011-2017 | 0,117 | 0,130 | 0,396 | 0,723 |
|  | Physiology or Medicine | | | |
| 1901-1910 | 1,035 | 1,743 | 0,268 | 0,000 |
| 1911-1920 | 1,154 | 0,000 | 0,000 | 0,124 |
| 1921-1930 | 0,276 | 0,387 | 0,819 | 0,096 |
| 1931-1940 | 0,142 | 1,025 | 1,092 | 1,303 |
| 1941-1950 | 0,000 | 0,116 | 0,807 | 1,426 |
| 1951-1960 | 0,096 | 0,420 | 0,493 | 2,046 |
| 1961-1970 | 0,581 | 0,268 | 1,112 | 1,623 |
| 1971-1980 | 0,181 | 0,079 | 0,527 | 1,578 |
| 1981-1990 | 0,000 | 0,033 | 0,394 | 1,202 |
| 1991-2000 | 0,000 | 0,314 | 0,071 | 1,036 |
| 2001-2010 | 0,168 | 0,076 | 0,605 | 0,739 |
| 2011-2017 | 0,117 | 0,033 | 0,198 | 0,723 |

Frequencies of Nobel laureates across the three career events (HD, PWR, NP), relative to GDP per capita (thousand US$, in 2011 prizes). The final period of 2011–2017 (NP) is weighted and thus comparable to earlier 10-year periods.
